# Insecticidal Properties of Erythritol on Four Tropical Tephritid Fruit Flies, *Zeugodacus cucurbitae*, *Ceratitis capitata*, *Bactrocera dorsalis*, and *B. latifrons* (Diptera: Tephritidae)

**DOI:** 10.3390/insects14050472

**Published:** 2023-05-16

**Authors:** Dong H. Cha, Dominick Skabeikis, Bong-Soo Kim, Jana C. Lee, Man-yeon Choi

**Affiliations:** 1 Daniel K. Inouye US Pacific Basin Agricultural Research Center, USDA Agricultural Research Service, 64 Nowelo St., Hilo, HI 96720, USA; dominick.skabeikis@usda.gov; 2 Horticultural Crops Research Unit, USDA Agricultural Research Service, 3420 NW Orchard Ave., Corvallis, OR 97330, USA; jana.lee@usda.gov; 3 Animal and Plant Quarantine Agency, Gimcheon 39660, Republic of Korea; bskim79@korea.kr

**Keywords:** non-toxic insecticide, non-nutritive sugar, oriental fruit fly, melon fly, Mediterranean fruit fly

## Abstract

**Simple Summary:**

Tephritid fruit flies are among the most destructive agricultural pests of fruits and vegetables worldwide and have global significance, introducing barriers to the trade of fresh tropical commodities. The melon fly, Mediterranean fruit fly, oriental fruit fly, and Malaysian fruit fly have entered and become established in Hawaii and have been making frequent incursions into agriculturally important states of the U.S. mainland, such as California and Florida. Bait sprays containing protein food bait plus an insecticide such as spinosad have been a major control method for these fruit flies. However, resistance to bait sprays has been reported. In this study, we evaluated the potential insecticidal effects of five different non-nutritive sugars on four species of fruit flies established in Hawaii. Erythritol alone or erythritol plus sucrose formulations have a significant negative impact on their survival, suggesting a potential use of erythritol as a non-toxic management tool for the control of tropical tephritid fruit flies.

**Abstract:**

Tephritid fruit flies are among the most destructive agricultural pests of fruits and vegetables worldwide and can impose trade barriers against the movement of fresh tropical commodities. Primary pre-harvest control methods for these flies rely on the spraying of conventional chemical insecticides or bait sprays. However, resistance to these control methods has been reported in fruit flies. Erythritol is a non-nutritive sugar alternative for human consumption, which has been tested and confirmed for its insecticidal properties against various insect pest species. In this study, using laboratory bioassays, we evaluated the insecticidal effect of erythritol alone or various erythritol formulations containing sucrose and/or protein on four tropical fruit fly species established in Hawaii (e.g., melon fly, Mediterranean fruit fly, oriental fruit fly, and Malaysian fruit fly). In addition, the effects of other non-nutritive hexose and pentose sugar alcohols, such as sorbitol, mannitol, and xylitol, were tested. Among the different standalone and combinatory treatments tested, 1M erythritol and a combinatory formulation of 2M erythritol + 0.5M sucrose appeared to be the most detrimental to the survival of all four species of tested flies, suggesting the potential of using erythritol as a non-toxic management tool for the control of tropical tephritid fruit flies.

## 1. Introduction

Tropical tephritid fruit flies are among the most destructive agricultural pests of fruits and vegetables worldwide. They are serious pests both in native and in established ranges and pose significant trade barriers that disrupt the export and import of fresh tropical commodities [1,2]. *Zeugodacus* (Bactrocera) *cucurbitae* (Coquillett) (melon fly), *Ceratitis capitata* (Wiedemann) (Mediterranean fruit fly), *Bactrocera dorsalis* (Hendel) (oriental fruit fly), and *B. latifrons* (Hendel) (Malaysian fruit fly) (Diptera: Tephritidae) entered Hawaii in 1895, 1907, 1945, and 1983, respectively [3,4], and have been making frequent incursions into agriculturally important states such as California, Florida, and Texas since 1954 [5]. Strong tropical fruit fly prevention, outbreak response, and quarantine programs are in place [2], which have prevented these flies from being established in the U.S. mainland. However, their invasion frequency has been on the rise in recent years and there is a critical need to improve the fruit fly management programs in the U.S.

Protein bait sprays laced with insecticides have been widely used to control these fruit flies in outbreak areas [6], in combination with surveillance traps, the male annihilation technique, and the sterile insect technique [1]. Malathion is an inexpensive contact organophosphate insecticide and has been a conventional choice for fruit fly bait sprays. However, its low residue tolerance in export markets and adverse effects on beneficial insects, non-target insects, and humans have caused a decline in the use of malathion in bait sprays [7]. The most prominent replacement of malathion bait has been a spinosad-based hydrolyzed protein bait (GF-120, Dow AgroSciences, Indianapolis, IN, USA) [7], which was initially developed by Moreno and Mangan [8]. With low mammalian toxicity and a reduced impact on natural enemies of spinosad [9], GF-120 has become the most widely used tephritid control product. However, GF-120 is expensive and needs to be ingested. Moreover, the almost exclusive use of GF-120 as a spray product for fruit fly control has led to the development of resistance in *Z. cucurbitae* and other fruit fly species [10,11].

Erythritol is a four-carboned sugar alcohol (= polyol) and the main ingredient of the natural sweetener Truvia®, which is a non-nutritive (zero caloric) for human consumption. Since erythritol was discovered to be toxic to *Drosophila melanogaster* [12], erythritol toxicity was later investigated with multiple insect groups: *D. suzukii* (Diptera: Drosophilidae) [13,14], *Bactrocera dorsalis* (Diptera: Tephritidae) [15], *Musca domestica* (Diptera: Muscidae) [16,17], *Stomoxys calcitrans* (Diptera: Muscidae) [18], *Aedes aegypti* (Diptera: Culicidae) [19,20], and *Cacopsylla pyricola* (Hemiptera: Psyllidae) [21].

The effects of erythritol have been evaluated alone or in combination with phagostimulative sugars (sucrose, sucralose) on some beneficial non-target species. This includes the honeybee *Apis mellifera* (Hymenoptera: Apidae), both larvae [22] and adults [23], a pupal parasitoid of *D. suzukii*, *Pachycrepoideus vindemiae* (Hymenoptera: Pteromalidae), the western yellowjacket *Vespula pensylvanica* (Hymenoptera: Vespidae) [22], and a predatory spider mite *Tetranychus urticae* (Trombidiformes: Tetranychidae) [24]. No apparent toxicity was observed in bees and yellowjackets and minimal impacts were observed on the parasitoid and predatory mites, which fared better than the target pest. It is important to understand the physiological action and toxicity of the insecticidal erythritol in insects in order to deliver it to target pests and avoid non-targets in the field.

The physiological action of erythritol has been suggested to be via osmotic imbalance between the hemocoel and the digestive system in the body. For example, in *D. suzukii*, the erythritol-fed fly cannot metabolize and convert the erythritol to nutritional carbohydrates [13], and thus needs to excrete the erythritol immediately through the digestive tract [25,26]. A large amount of water in the body is required to dilute or excrete unmetabolized erythritol, but the excretion process is slow, leading to the accumulation of excessive erythritol molecules in the hemolymph, which can increase osmotic pressure and dehydration, leading to eventual death.

In this study, we selected four tropical tephritid fruit fly species, *Z. cucurbitae*, *C. capitata, B. dorsalis*, and *B. latifrons*, and evaluated the effects of erythritol, other sugar alcohols, erythritol mixed with sucrose, and protein on their survival. Although erythritol has been previously shown to decrease the survivorship of *B. dorsalis* [15], it was evaluated with erythritol alone, not mixed or formulated with other sugars or proteins.

## 2. Materials and Methods

### 2.1. Insects

*Bactrocera dorsalis*, *B. latifrons*, *Z. cucurbitae*, and *C. capitata* pupae were obtained from the laboratory stock colonies maintained at USDA ARS Daniel K. Inouye U.S. Pacific Basin Agricultural Research Center in Hilo, HI. All four species were maintained on an artificial diet. Pupae were held in cubical screen cages (30 cm W × 30 cm D × 30 cm H; Bugdorm.com) housed in a dedicated rearing room maintained at 22.5 ± 1 °C, 55 ± 6% rh, and a 14:10 h L:D photoperiod until eclosion.

### 2.2. Sugars

All sugars tested in this study, meso-erythritol (99%), D-mannitol (98%), D-sorbitol (98%), xylitol (99%), and sucrose (99%), were purchased from Fisher Scientific (Hampton, NH, USA). 

### 2.3. Feeding Assay

The feeding arena consisted of a 1 L plastic deli container with a perforated lid in which the test flies were introduced at the start of the test. For Experiments 1 to 3, 10 adult flies (5 females and 5 males) from each species were placed into a bioassay arena upon emergence (Day 0), and for Experiments 4 and 5 were placed either newly emerged (Day 0) or 7-day-old (Day 7) flies. In the arenas, flies were offered one of the sugar treatments as listed below (10 flies from one of four fly species/treatment/arena). Sugar solutions were provided in 4 mL glass vials (15 × 45 mm) with a cotton wick inserted into the vial opening to absorb the liquid sugar. Vials were inserted into a hole cut into the side of the plastic container, cotton wick slightly angled down, and secured with a strip of tape. This ensured that the cotton wicks were constantly saturated with sugar solution and minimized variation in sugar availability due to evaporation. Vials were refilled when solution levels were low. For all experiments described below, each test was replicated five times and survival of flies was recorded each day for 2 weeks.

### 2.4. Experiment 1: Effect of Non-Nutritive Sugar on Fly Survival 

Upon emergence (Day 0), 10 adult flies (5 females and 5 males) were placed in a bioassay arena as described above and offered a 1M concentration of (1) erythritol, (2) mannitol, (3) sorbitol, (4) xylitol, (5) sucrose, or (6) water. Water was offered as a negative control and sucrose as a positive control. 

### 2.5. Experiment 2: Effect of Sucrose Addition on Fly Survival

In order to clarify whether the observed decrease in survivorship from erythritol and mannitol was from starvation or the insecticidal effects of non-nutritive sugars, combined solutions of 1M erythritol + 1M sucrose and 1M mannitol + 1M sucrose were offered to the flies. Thus, the treatments tested included (1) 1M erythritol + 1M sucrose, (2) 1M mannitol + 1M sucrose, (3) 1M erythritol, (4) 1M mannitol, and (5) 1M sucrose as a positive control.

### 2.6. Experiment 3: Effect of Different Sugar Concentrations on Fly Survival

After confirming the insecticidal properties of erythritol, we tested at what concentration erythritol combined with sucrose had the biggest negative impact on survival rate. Treatments included (1) 2M erythritol + 1M sucrose, (2) 2M erythritol + 0.5M sucrose, (3) 1M erythritol + 1M sucrose, (4) 1M erythritol + 0.5M sucrose, and (5) 0.5M erythritol + 0.5M sucrose.

### 2.7. Experiment 4: Effect of Protein Availability on Fly Survival

In this experiment, we tested whether protein feeding could affect erythritol’s insecticidal impact using two different age groups of flies. Day 0 flies were considered as young flies and Day 7 flies as old flies. For old flies, upon emergence, stock flies in the rearing cage were fed a diet of 0.5M sucrose solution, yeast hydrolysate, and water for 7 days. Treatments included (1) 2M erythritol + 0.5M sucrose, and (2) 2M erythritol + 0.5M sucrose + protein. Erythritol and sucrose were combined and dispensed from a vial. For protein treatment, ten grams of yeast hydrolysate was separately provided in a small plastic Petri dish on the bottom of the deli container for the treatments with protein.

### 2.8. Experiment 5: Effect of Water Availability on Fly Survival

In this experiment, we tested whether water ingestion could affect the impact of erythritol, using two different age groups of flies as described above (Day 0 and Day 7). Treatments included (1) 2M erythritol + 0.5M sucrose + water, (2) 2M erythritol + 0.5M sucrose, and (3) 0.5M sucrose. Erythritol and sucrose were combined and dispensed from a vial. For water treatment, additional water was separately provided in a 4 mL glass vial with a cotton wick inserted into the vial opening. 

### 2.9. Statistical Analyses

The cumulative proportion of flies alive within an arena over 2 weeks was analyzed for each species/experiment in a repeated measures generalized linear mixed model in SAS 9.4 (SAS Institute 2016). In Experiments 1–3, treatment, day, and treatment*day interactions were fixed effects, and treatment*replicate was the random subject effect being measured repeatedly. In Experiments 4–5, treatment, age, treatment*age, and day were fixed effects, and treatment*age*replicate was the random subject effect. A binomial or normal distribution was used depending on convergence. Treatment differences were separated by Tukey HSD.

## 3. Results

Survival of tested flies decreased over time (14 days) in all experiments, as expected (Day effect: all *p* < 0.0001).

### 3.1. Experiment 1: Effect of Non-Nutritive Sugar on Fly Survival 

**Figure 1:**
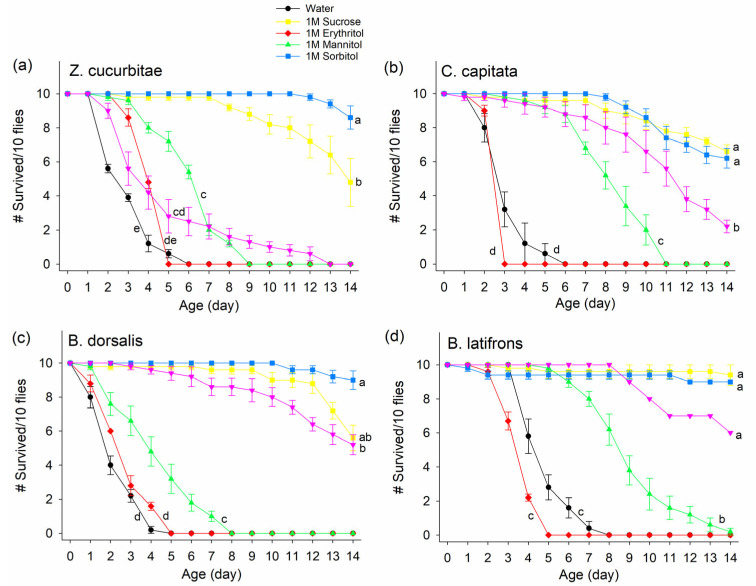
Survivorship of (**a**) *Zeugodacus cucurbitae*, (**b***) Ceratitis capitata*, (**c**) *Bactrocera dorsalis*, and (**d**) *Bactrocera latifrons* fed on 1M of sucrose, erythritol, mannitol, sorbitol, or xylitol over a 14 d period. Different letters indicate significant differences by Tukey HSD test at *p* < 0.05. # on Y-axis indicates “Number of”.

Among the non-nutritive sugars tested, erythritol and mannitol led to lower fly survival than the others (Figure 1). The survival rates of all four species were significantly lower when fed a solution of 1M erythritol, with all dead in 3–5 days among *Z. cucurbitae* (ZC) (Figure 1a), *C. capitata* (CC) (Figure 1b), *B. dorsalis* (BD) (Figure 1c), and *B. latifrons* (BL) (Figure 1d), than when fed other sugars: 1M mannitol, 1M sorbitol, 1M sucrose, or 1M xylitol (treatment effect ZC: F_5,24_ = 236.49, *p* < 0.0001; CC: F_5,24_ = 148.79, *p* < 0.0001; BD: F_5,24_ = 364.69, *p* < 0.0001; BL: F_5,20_ = 247, *p* < 0.0001; Figure 1). Among the sugar solutions, the survival rates of mannitol-fed flies were significantly lower than when flies were fed with sucrose. Mannitol-fed flies all died by day 9 for ZC, day 11 for CC, and day 8 for BD, and 80% of BL died by day 14. Sucrose-fed flies had 52% of ZC, 34% of CC, 44% of BD, and 6% of BL dead by day 14. There was no difference between xylitol and mannitol for *Z. cucurbitae*. The survival rate of mannitol-fed flies was greater than when flies were fed with either erythritol or water. Water-fed flies all died by day 6 for ZC, day 6 for CC, day 5 for BD, and day 8 for BL. The survival rates of flies given only water were slightly different by day 8, but not significantly different from the erythritol treatment. For *Z. cucurbitae*, the survivorship of sorbitol-fed flies was significantly greater than that of sucrose-fed flies (Figure 1a). The survival rates of xylitol-fed flies were significantly lower than those of sucrose- or sorbitol-fed flies, except for *B. latifrons* flies. 

### 3.2. Experiment 2: Effect of Sugar Concentration on Fly Survival

**Figure 2:**
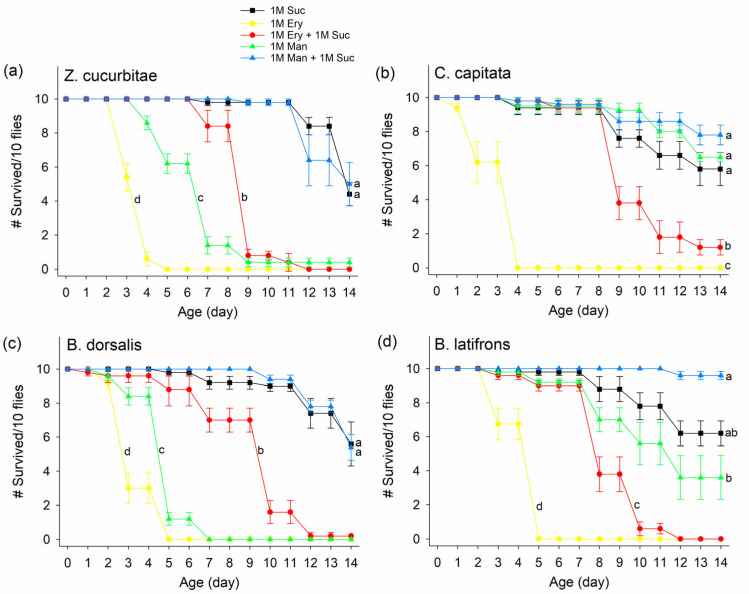
Survivorship of (**a**) *Zeugodacus cucurbitae*, (**b**) *Ceratitis capitata,* (**c**) *Bactrocera dorsalis*, and (**d**) *Bactrocera latifrons* fed on 1M sucrose (Suc), 1M erythritol (Ery), 1M Ery + 1M Suc, 1M mannitol (Man), and 1M Man + 1M Suc over a 14 d period. Different letters indicate significant differences by Tukey HSD tests at *p* < 0.05. Statistical tests were based on the probability of live flies. Means from cumulative live fly data, males and females combined, are shown. # on Y-axis indicates “Number of”.

The potential insecticidal effect of erythritol and mannitol was further evaluated by feeding in combination with sucrose, because erythritol and mannitol are considered zero-caloric sugars (Figure 2). For all four fruit fly species, feeding on 1M erythritol significantly reduced survival rates, with all flies dead in 4–5 days compared to sucrose-fed flies, where 56% of ZC, 42% of CC, 44% of BD, and 38% of BL died by day 14 (ZC: F_4,20_ = 161.4, *p* < 0.0001; CC: F_4,19_ = 94.3, *p* < 0.0001; BD: F_4,20_ = 161.43, *p* < 0.0001; BL: F_4,19_ = 64.97, *p* < 0.0001). The negative impact of erythritol on fly survival was still maintained when sucrose was mixed in the solution (open circle for mixture, all ZC died by day 12, 88% of CC died by day 14, almost all BD died by day 12, and all BL died by day 12), although the survival rates from 1M erythritol + 1M sucrose treatment were significantly greater than those from 1M erythritol-only treatment. Unlike erythritol, 1M mannitol + 1M sucrose solution was not toxic to all four fly species (Figure 2), although feeding 1M mannitol alone reduced the survivorship of *Z. cucurbitae* and *B. dorsalis* (Figure 2a,c).

### 3.3. Experiment 3: Effect of Different Sugar Concentrations on Fly Survival 

**Figure 3:**
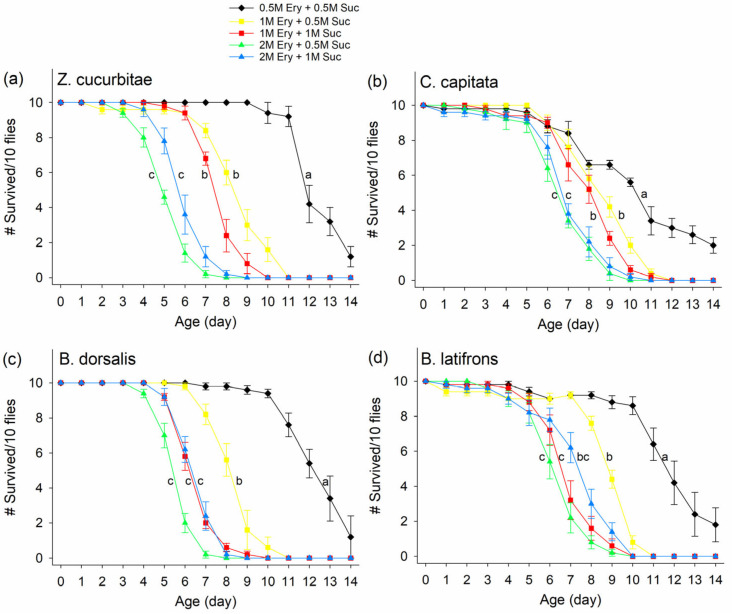
Survivorship of (**a**) *Zeugodacus cucurbitae*, (**b**) *Ceratitis capitata*, (**c**) *Bactrocera dorsalis*, and (**d**) *Bactrocera latifrons* fed on different doses of erythritol (Ery) and sucrose (Suc) over 14 d period. Different letters indicate significant differences by Tukey HSD tests at *p* < 0.05. Statistical tests were based on probability of live flies. Means from cumulative live fly data, male and female combined, are shown. # on Y-axis indicates “Number of”.

There was a significant effect of different erythritol concentrations on fly survival rate over 2 weeks (Figure 3, ZC: F_4,20_ = 152.93, *p* < 0.0001; CC: F_4,20_ = 39.63, *p* < 0.0001; BD: F_4,20_ = 132.43, *p* < 0.0001; BL: F_4,20_ = 37.36, *p* < 0.0001). Except for the flies fed 0.5M erythritol + 0.5M sucrose, when flies were fed with 1M or 2M erythritol combined with 0.5M or 1M sucrose, the survival rates of all four fruit fly species were significantly reduced, with the lowest survival at 2M erythritol + 0.5M sucrose treatment (all ZC died by day 8, CC by day 10, BD by day 8, and BL by day 10). Varying sucrose doses between 0.5M and 1M within an erythritol dose did not have a consistent impact on fly mortality, with only significant differences observed between 1M erythritol + 1M sucrose and 1M erythritol + 0.5M sucrose treatments for the survivorship of *B. dorsalis* and *B. latifrons* (Figure 3c,d; 1M erythritol + 1M sucrose vs. 1M erythritol + 0.5M sucrose; open square vs. solid square line). 

### 3.4. Experiment 4: Effect of Age and Protein Availability on Fly Survival

**Figure 4:**
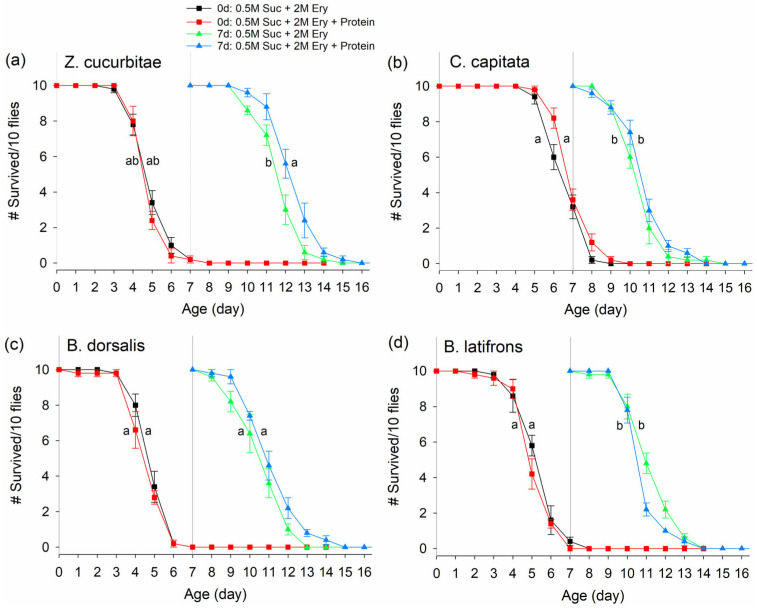
Survivorship of (**a**) *Zeugodacus cucurbitae*, (**b**) *Ceratitis capitata*, (**c**) *Bactrocera dorsalis*, and (**d**) *Bactrocera latifrons* fed on 2M erythritol (Ery) and 0.5M sucrose (Suc) with or without yeast hydrolysate (protein). Adult flies from two different age groups (0 d-old and 7 d-old flies) were monitored for survival over a 14 d period. Vertical line at 7 d indicates when 7 d-old flies began being fed with the treatments. Different letters indicate significant differences by Tukey HSD test at *p* < 0.05 based on days after feeding initiation. Statistical tests were based on the probability of live flies. Means from cumulative live fly data, males and females combined, are shown. # on Y-axis indicates “Number of”.

When Day 0 and Day 7 flies were fed 2M erythritol + 0.5M sucrose solution mixed with or without protein, fly survivorship was similar over 2 weeks (Figure 4; treatment effect ZC: F_1,16_ = 2.13, *p* = 0.1638; CC: F_1,16_ = 0.13, *p* = 0.7242; BD: F_1,16_ = 1.94, *p* = 0.1826, BL: F_1,16_ = 0.89, *p* = 0.3601). Age affected the survivorship of *C. capitata* and *B. latifrons*, as older flies died faster once feeding was initiated (Figure 4b,d; age effect CC: F_1,16_ = 15.34, *p* = 0.0012; BL: F_1,16_ = 14.76, *p* = 0.0014). Age did not affect the survivorship of *Z. cucurbitae* nor *B. dorsalis* (Figure 4a,c; Age effect ZC: F_1,16_ = 0.67, *p* =0.4237; BD: F_1,16_ = 1.96, *p* = 0.1801). For *Z. cucurbitae,* there was a marginal treatment*age interaction (Figure 4a; tr*age effect ZC: F_1,16_ = 4.03, *p* = 0.062). The survival rate of *Z. cucurbitae* from the Day 7 group slightly increased when fed 2 M erythritol + 0.5 M sucrose plus protein, compared with being fed only 2 M erythritol + 0.5 M sucrose (Figure 1a; solid triangle vs. open triangle lines).

### 3.5. Experiment 5: Effect of Water Availability on Fly Survival

**Figure 5:**
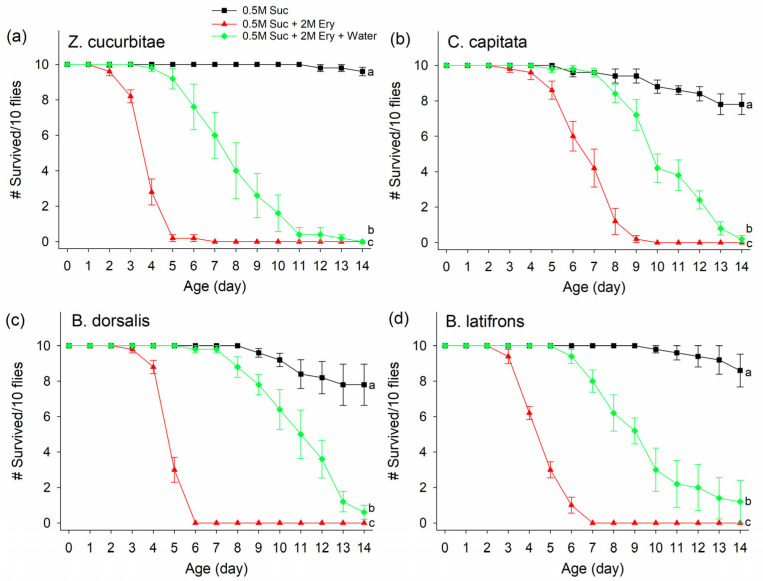
Survivorship of (**a**) *Zeugodacus cucurbitae*, (**b**) *Ceratitis capitata,* (**c***) Bactrocera dorsalis*, and (**d**) *Bactrocera latifrons* fed on a 2M erythritol (Ery) and 0.5M sucrose (Suc) mixture with or without additional water provided separately over a 14 d period. Different letters indicate significant differences by Tukey HSD test at *p* < 0.05. Statistical tests were based on the probability of live flies. Means from cumulative live fly data, males and females combined, are shown. # on Y-axis indicates “Number of”.

For all four species of flies tested with 2M erythritol + 0.5M sucrose, the insecticidal effect of erythritol was reduced when flies had access to additional water, although the insecticidal effect of erythritol was still observed from the treatment even with additional water supplemented (Figure 5; treatment effect ZC: F_2,24_ = 34.7, *p* < 0.0001; CC: F_2,24_ = 20.45, *p* < 0.0001; BD: F_2,24_ = 26.79, *p* < 0.0001; BL: F_2,24_ = 2121, *p* < 0.0001; Day 0 and Day 7 flies showed a similar result patten and only the Day 0 fly response is shown). Age affected *B. latifrons* as older ones died faster (age effect BL: F_1,24_ = 4.26, *p* = 0.05), but did not affect *Z. cucurbitae*, *C. capitata*, and *B. dorsalis* (age effect ZC: F_1,24_ = 1.14, *p* = 0.2954; CC: F_1,24_ = 0.02, *p* = 0.8776; BD: F_1,24_ = 0.07, *p* = 0.7925). For all flies, treatment*age interactions were insignificant (*p* > 0.05).

## 4. Discussion

In this study, we examined the insecticidal properties of four sugar alcohols individually (erythritol, mannitol, sorbitol, and xylitol) and different erythritol formulations containing sucrose and/or protein on the four tropical fruit fly species that are established in Hawaii. In the first experiment, all four species of flies tested died by day 8 of feeding either erythritol or water. This could be attributed to the insecticidal effect of erythritol, the lack of nutrition from erythritol consumption, or the absence of erythritol consumption by tested flies. Experiment 2, which tested the effect of the erythritol + sucrose combination, validated the insecticidal impact of erythritol on all four species of fruit flies tested, and, thus, in subsequent experiments, we used erythritol + sucrose formulations to ensure that flies consumed erythritol. These results are similar to those of previous studies with *D. suzukii*, where the erythritol + sucrose formulation induced more feeding than erythritol alone [13]. In our study, however, all four fly species also died when fed with mannitol solution only, with *Z. cucurbitae* being the most susceptible to mannitol. Xylitol, a five-carboned sugar, also reduced the survival of *Z. cucurbitae* but had minimal effect on *B. dorsalis* survival. The differences in the effect of xylitol between the two species could be from the different digestive or metabolic processes of the pentose molecules. Though not yet examined in insects, fewer carbon molecules are typically passed faster through intestinal membranes than hexose sugars [27,28]. The xylitol could be quickly absorbed and diffused through the midgut membrane in *Z. cucurbitae*, which may increase the osmotic pressure in the hemolymph before being excreted out. Further study is necessary to elucidate the possible physiological mechanisms. 

The toxicity of mannose or mannitol on insects represents a long-standing question. In a previous study, honeybees that ingested mannose died, which suggested a potential toxicity of mannose to honeybees [29], which was created by an imbalance of the enzymes involved in mannose metabolism. However, mannose toxicity was not found in *D. melanogaster* [30] and *D. suzukii* [13]. Notably, in this study, mannitol appeared to be potentially toxic to all four tephritid fruit flies. The physiological processes, including digestive and metabolic mechanisms, might differ between Drosophilidae and Tephritidae, indicating that the tephritid flies could have a problem controlling their osmotic pressure after they ingest mannitol. Further study is necessary to compare the structures of the digestive tracts and metabolic enzymes involved between the two families.

Interestingly, erythritol had minimal or no effect on all three hymenopteran species, as beneficial or non-target insects, examined in the laboratory and the field [22,23]. Insect digestive tracts are generally specialized for different feeding habits [31]. The digestive structure of hymenopteran insects might differ from that of other insect groups in terms of digesting and uptaking sugars from the midgut, transporting them into the hemolymph, and excreting them from the body. The relationship between physiological processes and specific digestive structure regarding the impacts of erythritol on dipteran and hymenopteran insects remains to be studied in the future.

When yeast hydrolysate, as a protein nutrient, was combined in the erythritol formulations, it did not affect fly survival in the four fly species tested. This could be because flies somehow did not uptake many proteins, or the proteins did not significantly contribute to the osmotic change in the fly hemolymph. Protein molecules are relatively larger than sugars, and the process of their breakdown and absorbance is slower than that of sugars [32]. Like mammals, proteins might not increase blood sugar levels in the fly, resulting in minimal or no impact on the osmolality of the hemolymph, resulting in mortality.

In all four tephritid species, the erythritol + sucrose formulation was detrimental compared with sucrose alone. Moreover, flies given the formulation with a separate water source lived longer than without separate water, similar to *D. suzukii* [33]. This further supports the hypothesis that non-nutritive sugars cause an osmotic imbalance in the tephritid flies and that erythritol molecules are accumulated in the hemolymph by a physiological difference between the uptake and excretion processes, as observed in *D. suzukii* [25,33]. The physiological mode of action underlying the insecticidal effect of erythritol, as a non-toxic management tool, will be critical to our understanding of how erythritol has insecticidal properties for some insect species but not others, such as the honeybee.

In conclusion, our results suggest that erythritol is a potential insecticide alternative that can be used to sustainably reduce tropical tephritid damage to their host fruit. Managing these flies is particularly challenging due to the limited availability of effective insecticides and the development of resistance to protein bait sprays. Although promising, additional studies are required, including (1) additional confirmatory trials with a large sample size of flies, (2) better characterization of the palatability of erythritol to fruit flies, (3) the development of effective formulations of erythritol and suitable delivery or application techniques such as sprayables or baits, and (4) the evaluation of the potential impact of erythritol on the host plant and fruit quality. 

## Data Availability

The data that support the findings will be made available through a Data Transfer Agreement following an embargo from the date of publication to allow for the commercialization of research findings.
